# Effect of chorioamnionitis on postnatal growth in very preterm infants: a population-based study in Japan

**DOI:** 10.1007/s00404-024-07757-y

**Published:** 2024-10-01

**Authors:** Takafumi Ushida, Rena Nosaka, Masahiro Nakatochi, Yumiko Kobayashi, Sho Tano, Kazuya Fuma, Seiko Matsuo, Kenji Imai, Yoshiaki Sato, Masahiro Hayakawa, Hiroaki Kajiyama, Tomomi Kotani, the Neonatal Research Network of Japan

**Affiliations:** 1 Department of Obstetrics and Gynecology, Nagoya University Graduate School of Medicine, 65 Tsurumai-cho, Showa-ku, Nagoya, 466-8550 Japan; 2 Division of Reproduction and Perinatology, Center for Maternal-Neonatal Care, Nagoya University Hospital, Nagoya, Japan; 3 Anne Women’s Clinic, Nagoya, Japan; 4 Public Health Informatics Unit, Department of Integrated Health Sciences, Nagoya University Graduate School of Medicine, Nagoya, Japan; 5 Data Science Division, Data Coordinating Center, Department of Advanced Medicine, Nagoya University Hospital, Nagoya, Japan; 6 Division of Neonatology, Center for Maternal-Neonatal Care, Nagoya University Hospital, Nagoya, Japan

**Keywords:** Catch-up growth, Histological chorioamnionitis, Inflammation, Preterm birth

## Abstract

**Purpose:**

There is growing evidence that preterm infants born to mothers with chorioamnionitis (CAM) have increased risk of various neonatal morbidities and long-term neurological disorders; however, the effect of CAM on postnatal growth remains insufficiently investigated. This study evaluated the effect of histological CAM on postnatal growth trajectories in very preterm infants using a nationwide neonatal database in Japan.

**Method:**

A multicenter retrospective study was conducted using clinical data of 4220 preterm neonates who weighed ≤ 1500 g and were born at < 32 weeks of gestation between 2003–2017 (CAM group: n = 2110; non-CAM group: n = 2110). Z-scores for height and weight were evaluated at birth and 3 years of age. Univariable and multivariable analyses were conducted to evaluate the effect of histological CAM on ΔZ-scores of height and weight during the first three years with a stratification by infant sex and the stage of histological CAM.

**Results:**

Multivariable analyses showed that histological CAM was associated with accelerated postnatal increase (ΔZ-score) in weight (β coefficient [95% confidence interval]; 0.10 [0.00 to 0.20]), but not in height among females (0.06 [− 0.04 to 0.15]) and not in height and weight among males (0.04 [− 0.04 to 0.12] and 0.02 [− 0.07 to 0.11], respectively). An interaction analysis demonstrated no significant difference in the effect of histological CAM on the ΔZ-scores of height and weight during the first three years between male and female infants (height, *p* = 0.81; weight *p* = 0.25).

**Conclusions:**

Intrauterine exposure to maternal CAM contributes to accelerated postnatal weight gain in female preterm infants during the first three years.

**Supplementary Information:**

The online version contains supplementary material available at 10.1007/s00404-024-07757-y.

## What does this study add to the clinical work

**Table:** 

Intrauterine exposure to maternal chorioamnionitis contributes to accelerated postnatal weight gain in female preterm infants during the first three years. This study indicates that female infants born to mothers with chorioamnionitis may be at high risk for childhood obesity and impaired glucose tolerance in young adulthood, underscoring the need for monitoring the anthropometric trajectory after 3 years of age.	

## Introduction

Chorioamnionitis (CAM), characterized by an ascending infection of the fetal membranes, amniotic fluid, and placenta due to pathogenic cervicovaginal microorganisms passing through the maternal vagina into the uterus, is a significant concern in obstetrics and neonatology [1, 2]. Although the prevalence of CAM varies, depending on gestational week, study population, ethnicity, region, and diagnostic criteria, the reported prevalence ranges between 4–40%, with higher rates observed in preterm deliveries [3]. CAM is a major cause of preterm labor and premature rupture of membranes, leading to a range of potentially life-threatening health consequences in preterm neonates, including respiratory complications (e.g., respiratory distress syndrome and chronic lung disease), sepsis, and neurological sequelae (e.g., intraventricular hemorrhage, periventricular leukomalacia, and cerebral palsy) [1, 4, 5].

However, recent studies have gathered sufficient evidence indicating a heightened lifetime susceptibility to neurodevelopmental, metabolic, and allergic disorders among offspring exposed to maternal immune activation (MIA) in utero [1, 6–9]. MIA refers to a maternal immune system response triggered by various immune stimuli such as autoimmune disorders, environmental factors, and infections, including CAM [8, 9]. Preclinical animal studies have shown that offspring exposed to MIA (e.g., lipopolysaccharide and poly(I:C)) in utero had altered metabolic pathways and glycemic regulation, leading to excess visceral and subcutaneous fat deposition [7, 10, 11]. However, clinical studies addressing the association between CAM exposure in utero and increased risk of metabolic disorders in humans have not yet been reported.

Although numerous studies have explored the association between CAM and various neonatal morbidities [1, 4, 12, 13], its effect of CAM on postnatal growth, especially in preterm infants, remains unclear. To date, only a few studies have addressed this issue [14, 15]. Therefore, the primary objective of this study was to investigate the effects of histological CAM on postnatal growth trajectories in very preterm infants at 3 years of age, using a nationwide neonatal database in Japan. Additionally, we sought to evaluate the potential sex-specific differences in the association between CAM and postnatal growth. Understanding the association between histological CAM and postnatal growth may offer valuable insights into the mechanisms underlying the adverse effects of intrauterine inflammation on long-term metabolic alterations in the offspring.

## Methods

### Study design and population

This retrospective cohort study used clinical data collected from the Neonatal Research Network of Japan (NRNJ), comprising preterm infants born in approximately 200 neonatal intensive care units (NICUs) between 2003–2017. The NRNJ is a collaborative network that was established to facilitate studies on neonatal medicine in Japan [16]. The NRNJ aims to improve the quality of neonatal care and establish optimal treatment strategies by collecting and sharing clinical data from NICUs across Japan. In the NRNJ database, preterm infants who weighed ≤ 1500 g and were born at 22–31 weeks of gestation were included in the analysis. Approximately 3000–4000 infants are registered in this database annually, equivalent to 65% of very low birth weight neonates born in Japan [16]. Infants from multiple pregnancies, those with major congenital abnormalities, and transferred from other facilities, neonatal deaths that occurred during their stay in the NICUs, as well as infants from mothers with hypertensive disorders of pregnancy, pre-existing diabetes mellitus or gestational diabetes mellitus, and those categorized as small or large for their gestational age, and infants with missing data on maternal and neonatal characteristics were excluded from the study. Additionally, infants without follow-up data at three years of age were excluded.

Written informed consent for registration in this database was obtained from the parents of the infants at each facility. However, in accordance with approvals from the ethical committees of individual facilities, informed consent was not obligatory and was waived in some facilities because all clinical data were anonymized. This study was approved by the Institutional Ethics Committee of Nagoya University Hospital (approval number: 2018–0026), and authorization for data usage was granted by the Japan Neonatal Network Executive Committee.

### Data collection

Demographic and clinical data were obtained from the NRNJ database center after anonymization. Maternal and neonatal clinical information included maternal age, gestational week at delivery, parity, delivery mode, histological CAM, antenatal corticosteroid treatment, infant sex, birth height, birth weight, and short-term neonatal outcomes at the time of NICU discharge. Histological CAM is diagnosed by histopathological examination of placental tissues, including neutrophil infiltration of the placental membranes, according to the Blanc classification (subchorionic [stage I], chorionic [stage II], and amniotic membrane [stage III]) [17, 18].

### Assessment of anthropometric measurements

Anthropometric measurements were obtained at birth and at a chronological age of 3 years, and assessed using Z-scores for weight and height. The Z-scores were calculated based on age- and sex-specific reference standards using software provided by the Japanese Society for Pediatric Endocrinology [19]. The software was developed using the LMS method [20]. The formula for the Z-scores is as follows: Z-scores = [(X/M) L − 1]/(L × S), where X is measured as heigh or weight values, M is median, L (Lambda) is asymmetry value, and S (Sigma) is variation coefficient. Z-scores enable a more precise assessment of infant growth than percentiles [21, 22].

### Statistical analysis

Statistical analyses were conducted using the SPSS 29 software (SPSS Inc., Chicago, IL, USA) and SAS version 9.4 (SAS Institute Inc., Cary, NC, USA). Demographic and clinical characteristics of the study population are summarized using descriptive statistics. Data are presented with mean ± standard deviation (SD), median (interquartile), or number (%). A total of 5263 infants who were followed-up at 3 years of age were randomly divided into two groups, CAM (n = 2110) and non-CAM (n = 2110), at a ratio of 1:1 after stratification by gestational age at birth and infant sex. The Z-scores of height and weight at 3 years of age were compared between the CAM and non-CAM groups and among the four groups (CAM stages I–III and non-CAM) using one-way analysis of variance or the Kruskal–Wallis test. Univariable and multivariable linear regression analyses were conducted to assess the effect of histological CAM on the postnatal growth (ΔZ-scores between birth and 3 years of age) of height and weight, with adjustment for two types of confounding factors (#1 [prenatal and early postnatal factors]: maternal age, gestational week at delivery, parity, mode of delivery, antenatal corticosteroid treatment, and Z-score of birth height [or weight], #2 [prenatal, early postnatal, and postnatal factors at NICU discharge]: maternal age, gestational week at delivery, parity, mode of delivery, antenatal corticosteroid treatment, Z-score of birth height [or weight], chronic lung disease, intraventricular hemorrhage [grade III/IV], periventricular leukomalacia, sepsis, necrotizing enterocolitis, patent ductus arteriosus banding, late-onset circulatory collapse, and total parenteral nutrition). Interaction analysis was performed to explore potential sex-specific differences in the association between histological CAM and postnatal growth using multivariate analyses adjusted for two types of confounding factors (#1 [prenatal and early postnatal factors] and #2 [prenatal, early postnatal, and postnatal factors at NICU discharge]). To confirm the consistency of the results, a sensitivity analysis that excluded cases with histological CAM stage I was conducted (CAM stages II/III vs. non-CAM) because the clinical significance of histological CAM stage I in neonatal outcomes is controversial [23, 24].

## Results

**Fig. 1:**
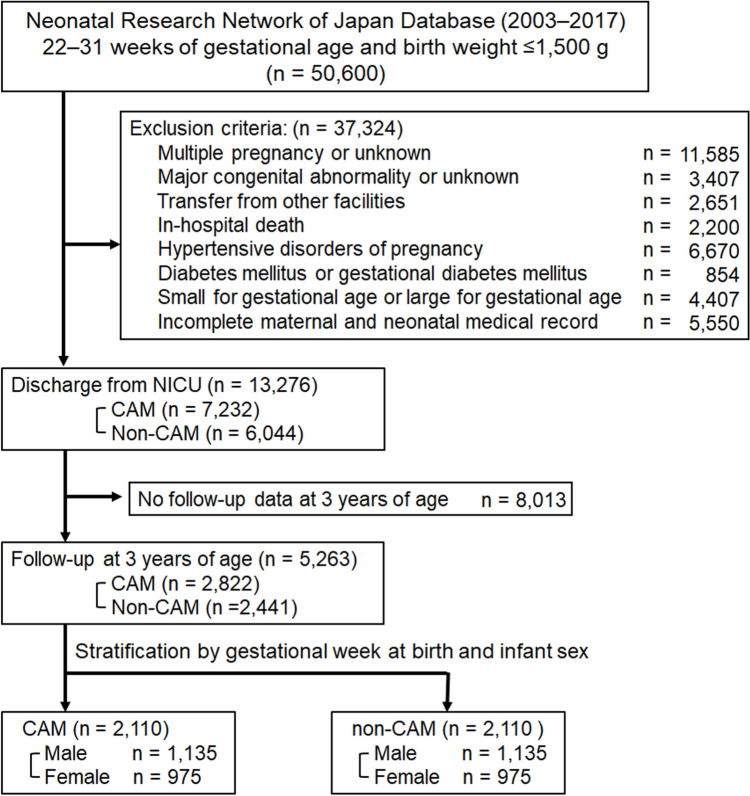
Flowchart of this study population. Clinical data of 50,600 preterm infants born at 22–31 gestational weeks, with birth weight ≤ 1500 g between 2003–2017 were obtained from the NRNJ database. A total of 5263 infants who underwent physical assessment at birth and 3 years of age were randomly divided into two groups, CAM (n = 2110) and non-CAM (n = 2110), at a ratio of 1:1 after stratification by gestational age at birth and infant sex. *SGA*, small for gestational age; *LGA* large for gestational age, *NICU* neonatal intensive care unit, *CAM* chorioamnionitis

The cohort study comprised 50,600 very preterm infants weighing ≤ 1500 g during the study period in the NRNJ database. A total of 5263 infants who underwent physical assessment at birth and 3 years of age (CAM, n = 2822; non-CAM, n = 2441) were randomly divided into two groups, CAM (n = 2110) and non-CAM (n = 2110), at a ratio of 1:1 after stratification by gestational age at birth and infant sex (Fig. 1). Table 1 summarizes the demographic and clinical characteristics of the study population. There were no significant differences in maternal age, gestational week, parity, infant sex, birth height, birth weight, or neonatal morbidities, except for chronic lung disease, sepsis, patent ductus arteriosus banding, and use of total parenteral nutrition. Mothers with histological CAM were less likely to deliver via cesarean section and more likely to receive antenatal corticosteroid treatment. Supplementary Table 1 summarizes the demographic and clinical characteristics of the study population before stratification according to gestational age at birth and infant sex.

**Table 1:** Maternal and neonatal baseline characteristics in the CAM and non-CAM groups after stratification by gestational age and infant sex

Variables	CAM	Non-CAM	*p*-value
	(n = 2110)	(n = 2110)	
*Maternal characteristics*			
Maternal age (years)	31.2 ± 5.2	30.9 ± 5.3	0.25
Gestational week at delivery (weeks)	27.3 ± 2.2	27.3 ± 2.2	0.67
22–23 (male/female)	93/54	93/54	
24–25 (male/female)	243/218	243/218	
26–27 (male/female)	348/263	348/263	
28–29 (male/female)	326/306	326/306	
30–31 (male/female)	125/134	125/134	
Primiparity	984 (46.6%)	962 (45.6%)	0.50
Cesarean section	1289 (61.1%)	1556 (73.7%)	< 0.01
Histological CAM			
Stage I	464 (22.0%)	N/A	
Stage II	618 (29.3%)	N/A	
Stage III	1028 (48.7%)	N/A	
ACS treatment	1414 (67.0%)	1163 (55.1%)	< 0.01
Neonatal characteristics			
Male	1135 (53.8%)	1135 (53.8%)	1.00
Birth height (cm)	34.9 ± 3.7	35.0 ± 3.7	0.20
Birth weight (g)	1013 ± 275	1016 ± 273	0.68
Chronic lung disease	627 (29.7%)	505 (23.9%)	< 0.01
IVH (grade III or IV)	88 (4.2%)	66 (3.1%)	0.07
PVL	73 (3.5%)	70 (3.3%)	0.80
Sepsis	196 (9.3%)	150 (7.1%)	0.01
Necrotizing enterocolitis	23 (1.1%)	21 (1.0%)	0.76
PDA banding	153 (7.3%)	189 (9.0%)	0.04
LCC	235 (11.1%)	247 (11.7%)	0.56
Total parenteral nutrition	1754 (83.1%)	1628 (77.2%)	< 0.01

Data are presented as mean ± standard deviation or number (%). *CAM*, chorioamnionitis, *ACS* antenatal corticosteroid, *IVH* intraventricular hemorrhage, *PVL* periventricular leukomalacia, *PDA* patent ductus arteriosus, *LCC* late-onset circulatory collapse, *N/A* not applicable

**Fig. 2:**
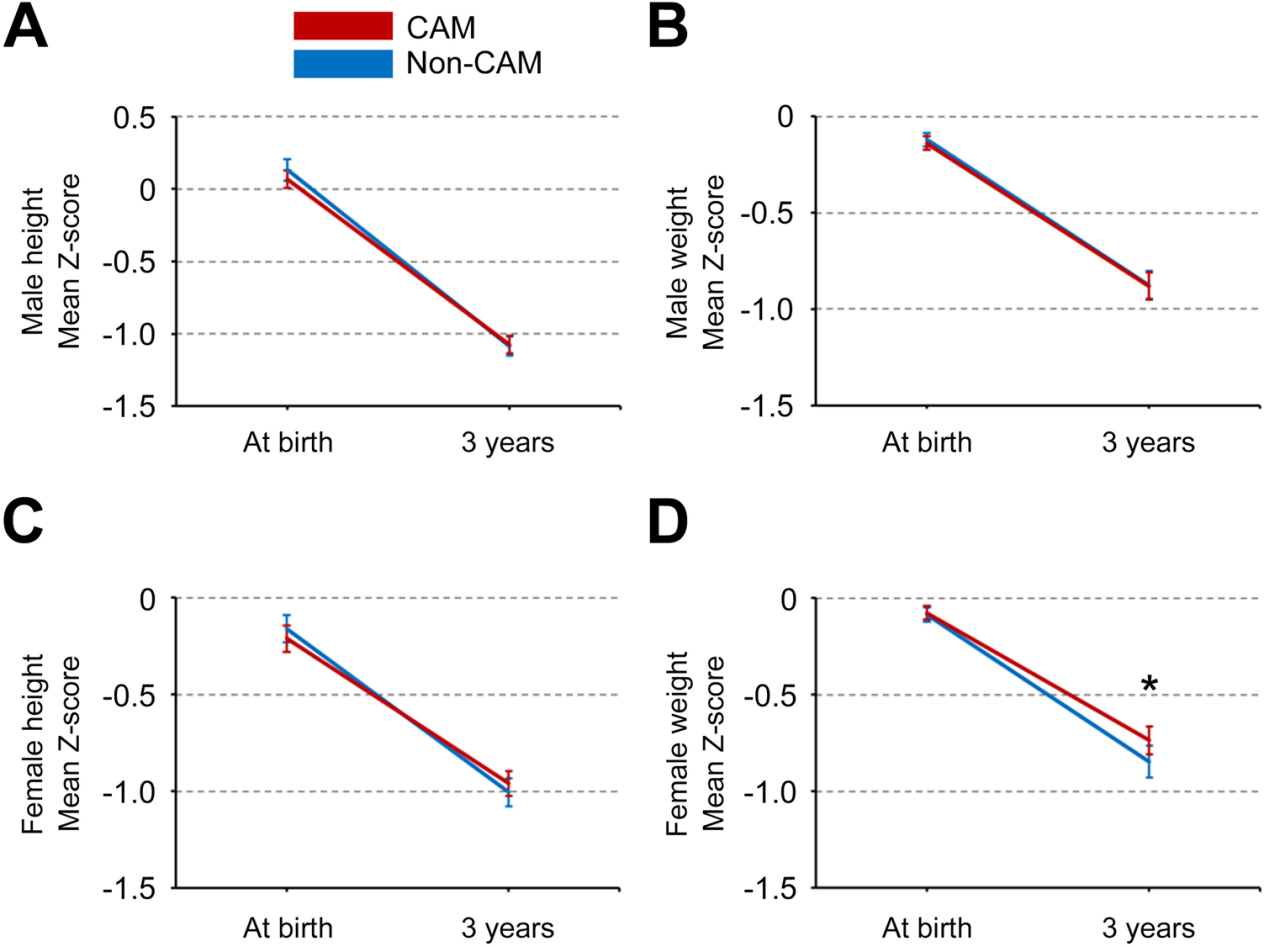
Trajectories of mean Z-scores for height and weight at birth and 3 years of age in the CAM and non-CAM groups. Mean Z-scores for height and weight with 95% confidence intervals in the CAM and non-CAM groups were evaluated (2A, male height; 2B, male weight; 2C, female height; 2D, female weight). *CAM* chorioamnionitis; *p < 0.05

**Fig. 3:**
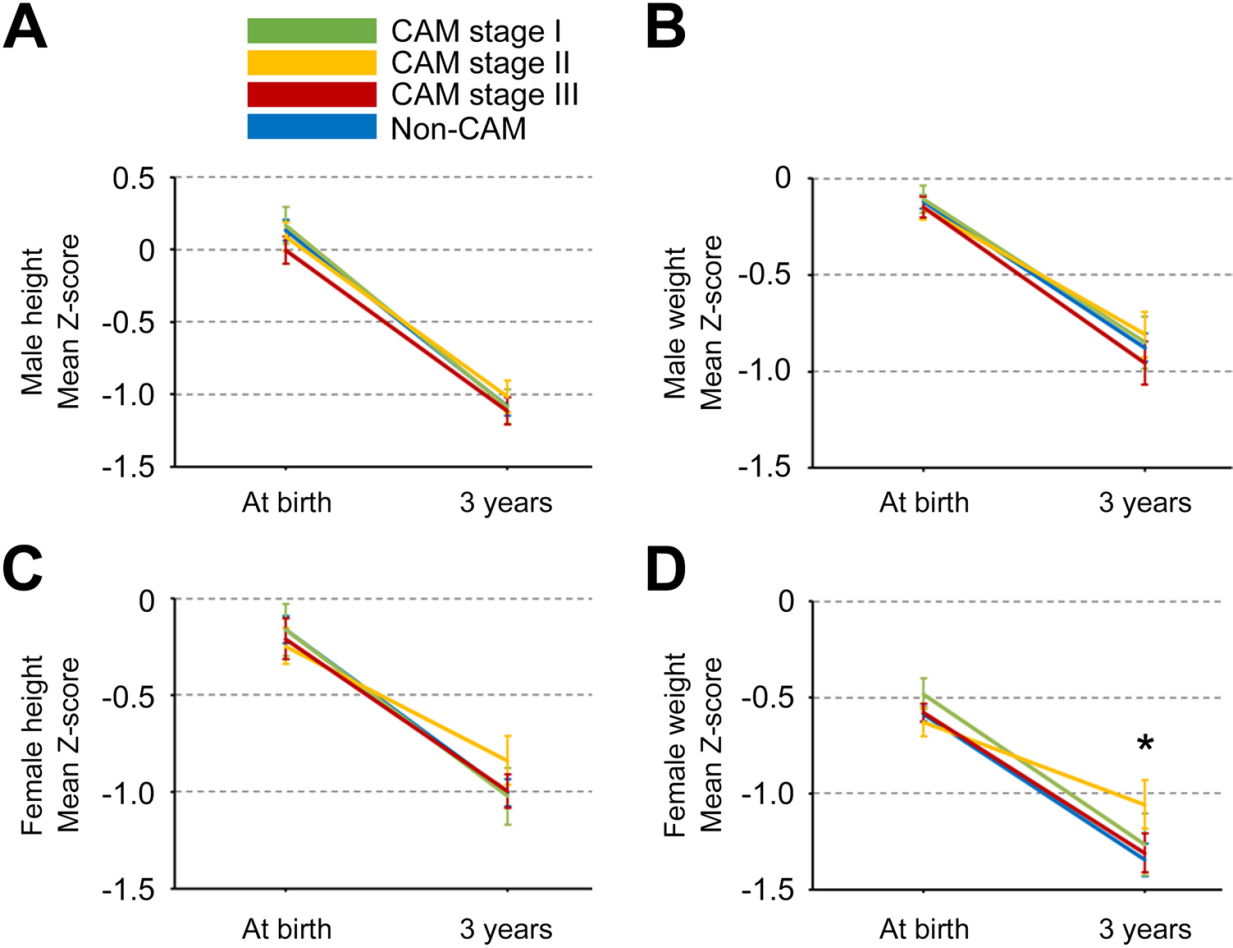
Trajectories of mean Z-scores for height and weight at birth and 3 years of age stratified by the stage of CAM. The mean Z-scores for height and weight with 95% confidence intervals (CIs) were evaluated according to CAM stage (3A, male height; 3B, male weight; 3C, female height; 3D, female weight). *CAM* chorioamnionitis; *p < 0.05

Supplementary Table 2 presents the physical assessment and Z-scores at birth and 3 years of age in the CAM and non-CAM groups. Figure 2 shows the trajectories of the mean Z-scores for height and weight during the first three years after birth in the CAM and non-CAM groups. No significant differences in mean Z-scores for height and weight at birth and 3 years of age were observed between the CAM and non-CAM groups, except for mean Z-scores for female weight at 3 years of age (*p* < 0.05). Figure 3 shows the trajectories of mean Z-scores for height and weight during the first three years after birth stratified by CAM stage. No significant differences in mean Z-scores for height and weight at birth and 3 years of age were observed among the four groups (non-CAM, and CAM stages I–III), except for mean Z-scores for female weight at 3 years of age (*p* < 0.05). Supplementary Fig. 1 shows the distribution of Z-scores at birth and ΔZ-scores during the first three years after birth in the CAM (red) and non-CAM (blue) groups. The distributions in the CAM and non-CAM groups were similar, and negative correlations between Z-scores at birth (X-axis) and ΔZ-scores for height and weight during the first three years (Y-axis) were observed in both males and females.

Univariable and multivariable linear regression analyses were conducted to assess the effect of histological CAM on the postnatal growth (ΔZ-scores) of height and weight during the first three years after birth (Table 2). Multivariable analysis (covariates #2) showed that histological CAM was associated with accelerated postnatal increase (ΔZ-score) in weight (β coefficient [95% confidence interval]; 0.10 [0.00 to 0.20]), but not in height in female infants (0.06 [− 0.04 to 0.15]) and not in height and weight in male (0.04 [− 0.04 to 0.12], 0.02 [− 0.07 to 0.11], respectively). A similar result was obtained in the multivariate analysis (covariate #1) (Table 2). A sensitivity analysis that excluded cases with histological CAM stage I was conducted (CAM stages II/III vs. non-CAM), which showed a significant association between histological CAM stages II/III and accelerated postnatal weight gain in female infants (covariates #1 and #2) (Table 3).

**Table 2:** Effect of histological chorioamnionitis on postnatal infant growth during the first three years after birth in the univariable and multivariable regression analyses

	Univariable	Multivariable #1	Multivariable #2
	β coefficient (95% CI)	β coefficient (95% CI)	β coefficient (95% CI)
Male height ΔZ-score	0.07 (− 0.05 to 0.20)	0.02 (− 0.06 to 0.11)	0.04 (− 0.04 to 0.12)
Male weight ΔZ-score	0.01 (− 0.09 to 0.12)	0.00 (− 0.09 to 0.09)	0.02 (− 0.07 to 0.11)
Female height ΔZ-score	0.10 (− 0.03 to 0.22)	0.05 (− 0.04 to 0.14)	0.06 (− 0.04 to 0.15)
Female weight ΔZ-score	0.10 (− 0.02 to 0.21)	0.10 (0.00 to 0.20)	0.10 (0.00 to 0.20)

The effect of histological chorioamnionitis on the postnatal increase (ΔZ-scores) in height and weight during the first three years after birth was evaluated using univariable and multivariable linear regression analyses. Multivariate analyses were adjusted for the following two types of covariates: #1 (prenatal and early postnatal factors), maternal age, gestational week at delivery, parity, mode of delivery, antenatal corticosteroid treatment, and Z-score of birth height (or weight); #2 (prenatal, early postnatal, and postnatal factors at NICU discharge): maternal age, gestational week at delivery, parity, mode of delivery, antenatal corticosteroid treatment, Z-score of birth height (or weight), chronic lung disease, intraventricular hemorrhage (grade III/IV), periventricular leukomalacia, sepsis, necrotizing enterocolitis, patent ductus arteriosus banding, late-onset circulatory collapse, and total parenteral nutrition. Data are presented as coefficients (95% CI). *CI* confidence interval; *NICU* neonatal intensive care unit

**Table 3:** Effect of histological chorioamnionitis on postnatal infant growth during the first three years after birth in the univariable and multivariable regression analyses after exclusion of cases with histological chorioamnionitis stage I

	Univariable	Multivariable #1	Multivariable #2
	β coefficient (95% CI)	β coefficient (95% CI)	β coefficient (95% CI)
Male height ΔZ-score	0.11 (− 0.02 to 0.24)	0.05 (− 0.05 to 0.14)	0.07 (− 0.02 to 0.16)
Male weight ΔZ-score	0.01 (− 0.10 to 0.13)	0.02 (− 0.08 to 0.12)	0.04 (− 0.05 to 0.14)
Female height ΔZ-score	0.12 (− 0.02 to 0.26)	0.07 (− 0.03 to 0.16)	0.07 (− 0.03 to 0.17)
Female weight ΔZ-score	0.13 (0.01 to 0.25)	0.12 (0.01 to 0.23)	0.12 (0.01 to 0.22)

The effect of histological chorioamnionitis on the postnatal growth (Δ Z-scores) of height and weight during the first 3 years after birth was evaluated using univariable and multivariable linear regression analyses. Multivariate analyses were adjusted for the following two types of covariates: #1 (prenatal and early postnatal factors), maternal age, gestational week at delivery, parity, mode of delivery, antenatal corticosteroid treatment, and Z-score of birth height (or weight); #2 (prenatal, early postnatal, and postnatal factors at NICU discharge): maternal age, gestational week at delivery, parity, mode of delivery, antenatal corticosteroid treatment, Z-score of birth height (or weight), chronic lung disease, intraventricular hemorrhage (grade III/IV), periventricular leukomalacia, sepsis, necrotizing enterocolitis, patent ductus arteriosus banding, late-onset circulatory collapse, and total parenteral nutrition. Data are presented as coefficients (95% CI). *CI* confidence interval; *NICU* neonatal intensive care unit

Interaction analysis demonstrated no significant difference in the effect of histological CAM on the ΔZ-scores of height and weight between male and female infants (covariates #1: height *p* = 0.64, weight *p* = 0.16; covariates #2: height* p* = 0.81, weight *p* = 0.25) (Table 4).

**Table 4:** Interaction between infant sex and chorioamnionitis on postnatal infant growth during the first 3 years after birth

	Multivariable #1		Multivariable #2	
	β coefficient (95% CI)	*p*-value	β coefficient (95% CI)	*p*-value
(Height) Infant sex × CAM	− 0.03 (− 0.15 to 0.09)	0.64	− 0.02 (− 0.14 to 0.11)	0.81
(Weight) Infant sex × CAM	− 0.09 (− 0.23 to 0.04)	0.16	− 0.08 (− 0.21 to 0.05)	0.25

The interaction between infant sex and histological chorioamnionitis on the postnatal growth (ΔZ-scores) of height and weight during the first 3 years after birth was evaluated using multivariable analyses. Multivariable analyses were adjusted for two types of covariates as follows: #1 (prenatal and early postnatal factors): maternal age, gestational week at delivery, parity, mode of delivery, antenatal corticosteroid treatment, Z-score of birth height (or weight), and infant sex × histological chorioamnionitis. #2 (prenatal, early postnatal, and postnatal factors at NICU discharge): maternal age, gestational week at delivery, parity, mode of delivery, antenatal corticosteroid treatment, Z-score of birth height (or weight), chronic lung disease, intraventricular hemorrhage (grade III/IV), periventricular leukomalacia, sepsis, necrotizing enterocolitis, patent ductus arteriosus banding, late-onset circulatory collapse, total parenteral nutrition, and infant sex × histological chorioamnionitis. Data are presented as coefficients (95% confidence interval [CI]). *CAM* chorioamnionitis; *CI* confidence interval

## Discussion

In this multicenter retrospective study, we evaluated the association between histological CAM and postnatal growth during the first three years after birth using a nationwide neonatal database in Japan. The main finding of this study was that female infants born to mothers with histological CAM exhibited accelerated postnatal weight gain during early childhood. Although there is growing evidence of an increased risk of neurological disorders in later life due to intrauterine exposure to CAM and preclinical findings of metabolic alterations in an MIA animal model [5, 7, 9, 10], to our knowledge, this is the first study to demonstrate that CAM exposure contributes to accelerated postnatal growth in humans. Accelerated growth, known as catch-up growth, in early childhood is associated with childhood obesity and subsequent risk of diabetes mellitus [25, 26]. Thus, our study suggests that female infants born to mothers with histological CAM may be at high risk of obesity and impaired glucose tolerance in young adulthood.

Our findings are inconsistent with those of two previous studies that evaluated the postnatal short- and medium-term growth of infants born to mothers with histological CAM. González et al*.* evaluated the postnatal growth of preterm neonates exposed to CAM during the first four weeks after birth [14]. The authors found no significant association between CAM levels and short-term postnatal growth. Mu et al*.* evaluated medium-term anthropometric growth during the first two years after birth [15]. The authors also found no significant association between histological CAM and medium-term postnatal growth. However, the sample sizes in these studies were quite small (n = 88 and 64, respectively); therefore, appropriate statistical evaluation could not be conducted due to insufficient participants.

Catch-up growth refers to the phenomenon in which infants who experience growth restriction early in life (e.g., in utero) subsequently undergo a rapid increase in growth, allowing them to “catch up” to a normal size for their age [27, 28]. Growth restriction can occur for various reasons, such as pregnancy complications (e.g., CAM, hypertensive disorders of pregnancy, and placental dysfunction), maternal undernutrition, preterm birth, and exposure to environmental substances [29, 30]. Although catch-up growth is generally considered beneficial for restoring health and normal development for infants, increasing evidence suggests that it may have long-term health implications, particularly, the risk of obesity, diabetes mellitus, and cardiovascular disorders will develop later in life [31–34]. The possible mechanisms of the increased risk of such disorders are as follows: first, according to the Developmental Origins of Health and Disease hypothesis, which originated from the David Baker’s theory, environmental factors can influence the programming of metabolic pathways and physiological systems during critical periods of fetal and early postnatal development [35]. A fetus with growth restriction may adapt its metabolism and physiology to survive in a hostile environment. However, if the condition improves later (e.g., after birth), these adaptations may lead to metabolic changes that predispose individuals to obesity and diabetes in adulthood. Second, epigenetic modifications can affect gene expression related to metabolic pathways (e.g., insulin signaling, adipogenesis, and lipid metabolism), increasing susceptibility to obesity and diabetes later in life [36–38]. Finally, catch-up and accelerated growth are often characterized by increased fat deposition, particularly visceral fat deposition. Excess visceral fat is strongly associated with insulin resistance, inflammation, and metabolic dysfunction, all of which are risk factors for obesity and type 2 diabetes [28].

Although the precise mechanistic link between intrauterine exposure to MIA and CAM and subsequent metabolic risks in the offspring remains unclear, metabolic alterations in later life may be associated with a complex interplay of maternal and fetal inflammatory responses within the intrauterine environment [6]. The inflammatory cascade initiated by MIA, including CAM, could have long-lasting effects on the offspring’s metabolic programming, potentially perturbing fetal development and disrupting nutrient supply, metabolic regulation, and endocrine signaling in multifaceted ways [6, 7, 10]. These disruptions may manifest as compromised fetal growth in utero and potentially extend into the postnatal period, altering the postnatal growth trajectory during critical early developmental phases.

Whether accelerated postnatal or catch-up growth occurs depends on various factors, including the timing, type, and period of insult during pregnancy, and infant sex [27, 28]. Regarding sex-specific differences, we found accelerated postnatal growth in weight in females but not in males. However, interaction analysis indicates that no significant sex difference was observed in the postnatal height and weight growth in during the first three years. We previously demonstrated that hypertensive disorders of pregnancy contribute to accelerated postnatal weight growth during the first three years after birth in both male and female infants born at < 32 weeks of gestation, indicating that the effect of infant sex on postnatal growth differs according to pregnancy complications [22]. Sex differences could be due to differences in hormonal influences (e.g., testosterone and estrogen), metabolic regulation, body composition, and genetic and epigenetic factors; however, the underlying mechanisms remain unclear.

The strengths of this study are as follows: first, few studies have evaluated the postnatal growth trajectories of infants born to mothers with CAM. Additionally, we demonstrated accelerated postnatal weight gain only in female child. Second, the sample size of this study was large and observation period was longer than that of previous studies [14, 15]. We also adjusted for covariates that may affect postnatal growth. In particular, gestational age at birth and Z-scores at birth are important factors that contribute to postnatal growth. Third, we performed interaction analysis to evaluate the effects of sex on postnatal growth.

This study has some limitations. First, the follow-up rate at 3 years of age was low (39.6%) because some children were transferred to other facilities, and some children without any complications may have completed the follow-up. Second, we excluded small for gestational age infants, large for gestational age infants, and infants born to mothers with hypertensive disorders of pregnancy, pre-existing diabetes mellitus, or gestational diabetes mellitus in order to eliminate the factors that can affect postnatal infants’ growth; however, there is a possibility that these exclusion criteria can lead to bias risk. Finally, we used the Blanc classification rather than the Amsterdam classification for the histological diagnosis of CAM; however, the Amsterdam classification system has been increasingly used recently [39].

In conclusion, our study highlights the significant association between histological CAM and accelerated postnatal weight gain in female infants born at < 32 weeks of gestation. This finding indicates that female infants born to mothers with histological CAM may be high risk for childhood obesity and impaired glucose tolerance in young adulthood, underscoring the need for further investigation to evaluate the long-term effect of histological CAM on risk of metabolic disorders. Additionally, our study indicates the need for monitoring the anthropometric trajectory after 3 years of age.

## Supplementary Information

Below is the link to the electronic supplementary material.

Supplementary file1 (TIF 23579 KB)

Supplementary file2 (TIF 23579 KB)

## Data Availability

Data that support the findings of this study are available from the corresponding author (TU) upon reasonable request and with permission from the Neonatal Research Network of Japan.
