# Supplementary material for: Effect of chorioamnionitis on postnatal growth in very preterm infants: a population-based study in Japan
Source: Arch Gynecol Obstet. 2024 Oct 1;311(5):1321–30. doi: 10.1007/s00404-024-07757-y (PMC12033191; doi:10.1007/s00404-024-07757-y)
Supplement: Supplementary file 1 — Supplementary file1 (TIF 23579 KB) [file 404_2024_7757_MOESM1_ESM.docx]

Supplementary Table 1. Maternal and neonatal baseline characteristics of the CAM and non-CAM groups before stratification by gestational age and infant sex

|  | CAM | Non-CAM |  |
| --- | --- | --- | --- |
| Variables | (n = 2,822) | (n = 2,441) | *p*-value |
| Maternal characteristics |  |  |  |
| Maternal age (years) | 31.3 ± 5.2 | 31.0 ± 5.3 | 0.03 |
| Gestational week at delivery (weeks) | 26.7 ± 2.3 | 27.7 ± 2.3 | <0.01 |
| 22–23 (male/female) | 159/170 | 93/54 |  |
| 24–25 (male/female) | 408/395 | 243/218 |  |
| 26–27 (male/female) | 461/338 | 348/263 |  |
| 28–29 (male/female) | 326/306 | 423/367 |  |
| 30–31 (male/female) | 125/134 | 217/215 |  |
| Primiparity | 1,290 (45.7%) | 1,109 (45.4%) | 0.84 |
| Cesarean section | 1,738 (61.6%) | 1,801 (73.8%) | <0.01 |
| Histological CAM |  |  |  |
| Stage I | 596 (21.1%) | N/A |  |
| Stage II | 806 (28.6%) | N/A |  |
| Stage III | 1,420 (50.3%) | N/A |  |
| ACS treatment | 1,884 (66.8%) | 1,341 (54.9%) | <0.01 |
| Neonatal characteristics |  |  |  |
| Male | 1,479 (52.4%) | 1,324 (54.2%) | 0.18 |
| Birth height (cm) | 34.1 ± 3.7 | 35.5 ± 3.7 | <0.01 |
| Birth weight (g) | 943 ± 280 | 1,057 ± 278 | <0.01 |
| Chronic lung disease | 1,003 (35.5%) | 522 (21.4%) | <0.01 |
| IVH (grade III or IV) | 130 (4.6%) | 68 (2.8%) | <0.01 |
| PVL | 86 (3.1%) | 82 (3.4%) | 0.52 |
| Sepsis | 299 (10.6%) | 157 (6.4%) | <0.01 |
| Necrotizing enterocolitis | 38 (1.4%) | 23 (0.9%) | 0.17 |
| PDA banding | 270 (9.6%) | 191 (7.8％) | 0.03 |
| LCC | 376 (13.3%) | 259 (10.6%) | <0.01 |
| Total parenteral nutrition | 2,405 (85.2%) | 1,836 (75.2%) | <0.01 |

Data are presented as mean ± standard deviation or number (%). CAM, chorioamnionitis; ACS, antenatal corticosteroid; IVH, intraventricular hemorrhage; PVL, periventricular leukomalacia; PDA, patent ductus arteriosus; LCC, late-onset circulatory collapse; N/A, not applicable.

Supplementary Table 2. Physical assessment and Z-scores at birth and 3 years of age in the CAM and non-CAM groups

|  | CAM | | Non-CAM | |
| --- | --- | --- | --- | --- |
|  | Male (n = 1,135) | Female (n = 975) | Male (n = 1,135) | Female (n = 975) |
| At birth |  |  |  |  |
| Height (cm) | 35.0 (32.5 to 37.6) | 35.0 (32.0 to 37.5) | 35.5 (32.5 to 38.0) | 35.0 (32.5 to 37.8) |
| Height (Z-score) | 0.06 (−0.56 to 0.66) | −0.22 (−0.76 to 0.33) | 0.09 (−0.44 to 0.68) | −0.18 (−0.74 to 0.36) |
| Weight (g) | 1,003 (812 to 1,246) | 999 (772 to 1,233) | 1,034 (812 to 1,252) | 999 (778 to 1,224) |
| Weight (Z-score) | −0.61 (−0.16 to 0.29) | −0.09 (−0.51 to 0.34) | −0.15 (−0.59 to 0.32) | −0.07 (−0.54 to 0.35) |
| At 3 years of age |  |  |  |  |
| Height (cm) | 90.4 (88.0 to 92.8) | 89.6 (87.3 to 92.0) | 90.3 (87.8 to 92.8) | 89.6 (87.0 to 92.0) |
| Height (Z-score) | −0.99 (−1.72 to −0.36) | −0.96 (−1.61 to −0.27) | −1.05 (−1.73 to −0.30) | −0.96 (−1.65 to −0.29) |
| Weight (kg) | 12.6 (11.5 to 13.5) | 12.3 (11.3 to 13.2) | 12.6 (11.6 to 13.6) | 12.2 (11.2 to 13.2) |
| Weight (Z-score) | −0.76 (−1.57 to −0.06) | −0.70 (−1.43 to 0.02) | −0.76 (−1.49 to −0.06) | −0.70 (−1.54 to 0.05) |

Data are presented as the median (interquartile range).
