# Supplementary figures and images for: Effect of chorioamnionitis on postnatal growth in very preterm infants: a population-based study in Japan
Source: Arch Gynecol Obstet. 2024 Oct 1;311(5):1321–30. doi: 10.1007/s00404-024-07757-y (PMC12033191; doi:10.1007/s00404-024-07757-y)

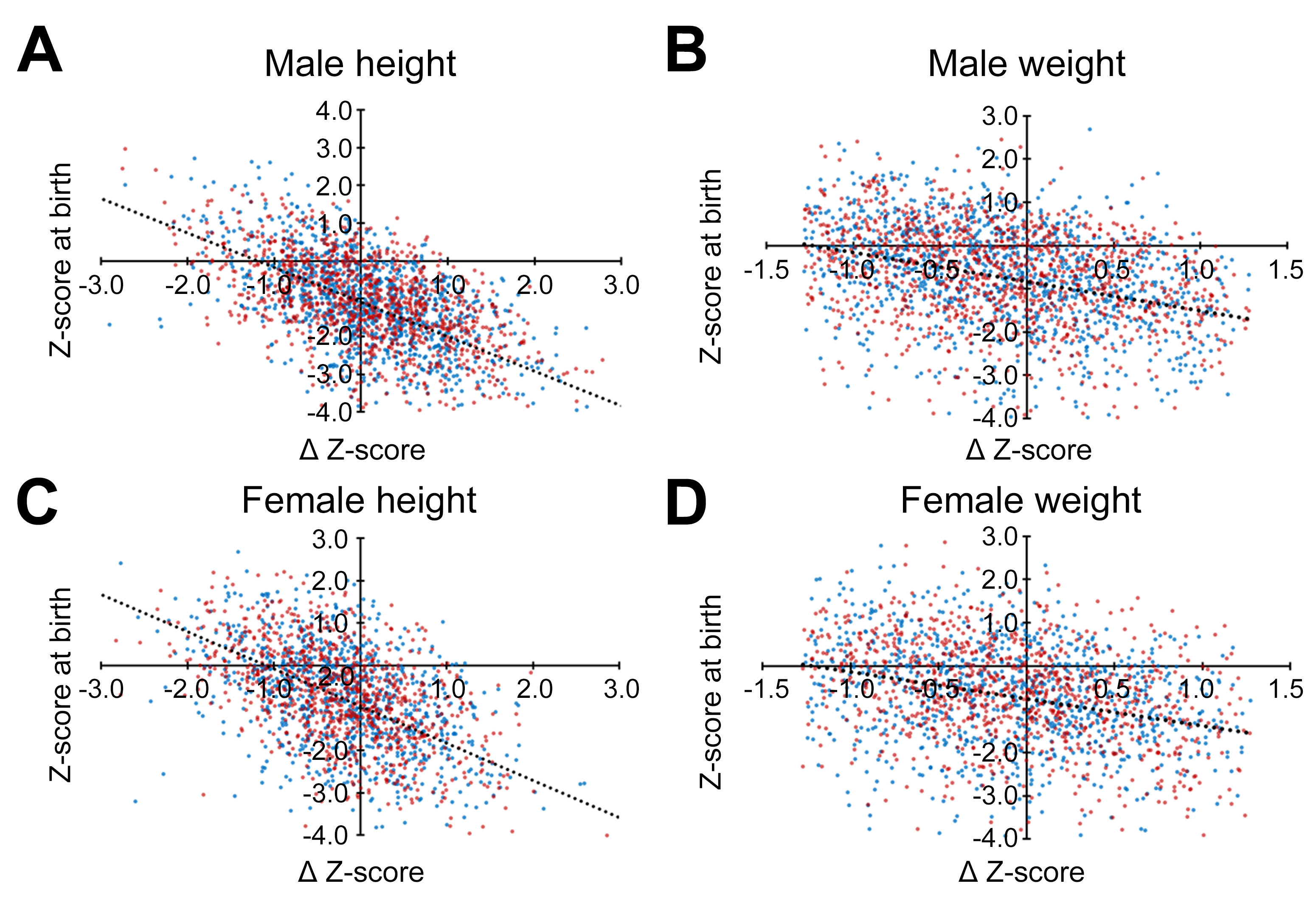

Supplement: Supplementary file 2 — Supplementary file2 (TIF 23579 KB) [file 404_2024_7757_MOESM2_ESM.tif]
